# Photothermal bleaching of nickel dithiolene for bright multi-colored 3D printed parts

**DOI:** 10.1038/s41467-022-35195-4

**Published:** 2023-02-03

**Authors:** Adekunle Olubummo, Lihua Zhao, Aja Hartman, Howard Tom, Yan Zhao, Kyle Wycoff

**Affiliations:** HP Labs, 1501 Page Mill Road, Palo Alto, CA 94304 USA

**Keywords:** Polymers, Organic molecules in materials science, Polymers

## Abstract

HP’s Multi Jet Fusion is a powder bed fusion 3D printing technology that utilizes a carbon-based radiation absorber in combination with a near infrared (NIR) light source to facilitate the fusion of polymer powder in a layer-by-layer fashion to generate 3D parts. Most available carbon-based and NIR radiation absorbers have an intrinsic dark color, which as a result will only produce black/gray and dark colored parts. However, there are many applications that require variable color, including prosthetics, medical models, and indicators, among others. To create white, bright colored, and translucent parts with MJF, a visibly transparent and colorless radiation absorber is required. In this paper, we designed an activating fusing agent (AFA) that contains a red, strong NIR absorbing dye that turns colorless after harvesting irradiation energy during the MJF 3D printing process and provide a bright colored part when working with other color agents.

## Abstract (web summary)

Most NIR radiation absorbers used in multi jet fusion 3D printing have an intrinsic dark color which prevents printing parts with variable colors. Here, the authors design an activating fusing agent containing a strong NIR absorbing dye that turns colorless after harvesting irradiation energy during the 3D printing process and provide a bright colored part when working with other color agents.

## Introduction

**Fig. 1:**
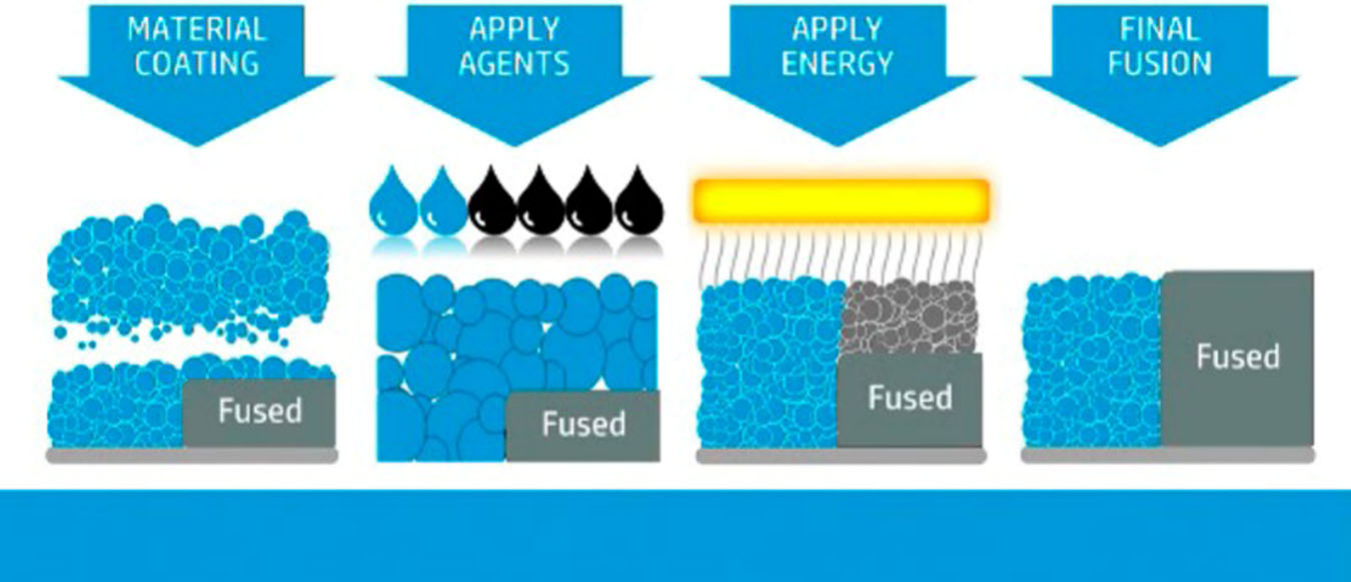
Diagram of additively manufacturing a part using the powder bed fusion-based HP Multi Jet Fusion technology. The MJF print process starts with a CAD model that is sliced to form a stack of 2D images. The first CAD model is printed using a black IR absorbing fusing agent onto the powder and then heated to melt using the broad area fusing lamps scanned vicinal to the bed. A detailing agent (DA) is jetted into areas where the fusing action will be reduced and into part boundaries to produce sharp and smooth edges for geometrically accurate parts through the process of evaporative cooling. A new layer of powder is spread on top of the previous layer and the process (print, fuse, spread) is repeated until the full object is formed.

3D printing is an advanced manufacturing method that fabricates parts through the conversion of 3D computer aided design (CAD) models into functional objects in a layer-by-layer method^1^. It offers the ability to produce parts rapidly, at low cost, short runs, and one-of-a-kind^2^, which has led to its adoption, shown by the additive manufacturing industry growth to 9.795 billion dollars in 2018^3^. There are many different additive manufacturing technologies including laser sintering, material extrusion, vat photopolymerization and powder bed fusion (PBF) processes^4^. The HP Multi Jet Fusion (MJF) technology is an example of PBF additive manufacturing. The MJF print process starts, like many AM processes, with a CAD model that is sliced to form a stack of 2D images. The first CAD model 2D geometry slice image is printed using a black IR absorbing fusing agent onto the powder and then heated to melt using the broad area fusing lamps scanned vicinal to the bed^5^. A detailing agent (DA) is jetted into areas where the fusing action will be reduced and into part boundaries to produce sharp and smooth edges for geometrically accurate parts through the process of evaporative cooling. A new layer of powder is spread on top of the previous layer and the process (print, fuse, spread) is repeated until the full object is formed as shown in Fig. 1. MJF technology offers speed, quality, strength, and novel functionalities along with the unique ability to produce parts with controllable physical and functional properties on the voxel level within a part^5^. The black IR absorbing fusing agent is mostly based on carbon black and, as a result, the 3D printed object produced is black or gray in color. This makes it very hard or impossible to produce white and colored parts with high color gamut without significant post processing, which limits their use in applications like prosthetics, medical models, sportswear, and indicators. The prosthetic market was valued at 6.11 billion dollars in 2020 and expected to have a compound annual growth rate of 4.25% over the next 8 years^6^. There are other 3D printing technologies, like fused deposition modeling (FDM), Selective Laser Sintering (SLS), Digital Light processing (DLP), Multi Jet Jetting (MJP), and Poly-jet which can produce white and colored parts.

To print white and brightly colored parts with the current MJF system, a fusing agent with strong NIR absorption coupled with low absorption in the visible range is required. The fusing agent should be easily formulated into an aqueous thermal inkjet compatible ink and tunnelable to the spectrum that matches with the emission spectrum on the IR source. This type of material is difficult to come by, as most available NIR dyes are colored, plagued with poor thermal properties, and have poor stability in aqueous solutions^7,8^. This makes it challenging to address markets where white, variable color, and translucency are important, like the prosthetics market. Gerasimos et al.^9^ demonstrate the use of gold nanorods (GNRs) as nanoengineered photothermal sensitizers, allowing the production of white or brightly colored 3D parts via selective laser sintering with low power diode lasers to reduce polymer degradation. The nanocomposite powders and photothermal sensitizer solutions were mechanically mixed with polyamide (PA12) powder to produce a nanocomposite for 3D printing. Objects were then formed using SLS. However, the plasmon resonance of the GNRs is strongly dependent on the size and shape of the nanoparticle, and so it is crucial that these properties are not significantly altered by either the mixing process or the sintering of the polymer powder during printing, which limits the scaling up of the process. In this paper, we present a method that enables printing white or intrinsic-colored 3D objects with MJF using an activating fusing agent (AFA) that contains a NIR absorbing dye that is red when printed and after harvesting irradiation energy is then bleached during the 3D printing process^10^. AFA is an aqueous based thermal ink jet formulation containing nickel bis(dithiolene) as the active NIR absorbing material. Nickel bis(dithiolene) complexes are important NIR dyes because of their unique properties, such as photostability, air-stability, thermal stability, intense absorption in the NIR region, easy adjustment of the absorption range with polar solvents, and high electron mobility^11^. These unique properties have enabled their application in thermal imaging, optoelectronics, photography, lithography, Q-switch absorber of a laser, optical switching, and antioxidant for polymers^12–14^.

**Fig. 2:**
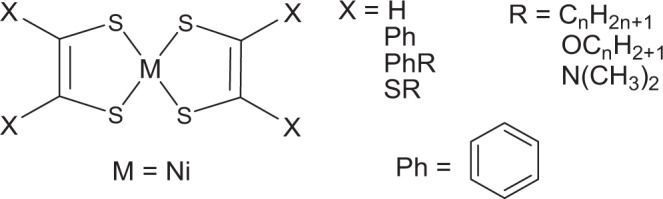
General structure of nickel bis(dithiolene) complex used for activating fusing agent. Unsaturated bidentate Dithiolene ligands, in which the two donor atoms are sulfur.

Nickel bis(dithiolene) complexes of the general structure shown in Fig. 2 exhibit strong absorption in the 600–1600 nm region of the electromagnetic spectrum and are highly soluble in nonpolar solvents like toluene and chloroform. The strong NIR absorption observed in nickel dithiolene is due to the electron delocalization about the dithiolene ring and the interaction of the delocalized electrons with the empty d-orbitals of the metal center^15^. Wang et al.^16^ reported the synthesis of colorless metallodithiolene oligomers and polymers with intense near- and mid-infrared absorption. The complex polymer is readily soluble in common organic solvents and shows remarkably broad and intense absorption in the entire near- and mid-infrared spectral region (800 nm–25 mm). The films of these metallodithiolene materials exhibit good visual transparency or extremely weak absorption in the visible region, making them potentially useful as excellent colorless infrared absorbers. However, in-depth study and applications of metallodithiolene polymers are seriously hindered by poor solubility and incomplete characterizations^17^. Here we used a commercially available nickel bis(dithiolene) complex of the general structure shown in Fig. 2 which can be easily reduced via electron transfer reaction, and converts into soluble mono or colorless dianionic form^18^.

## Results

**Fig. 3:**
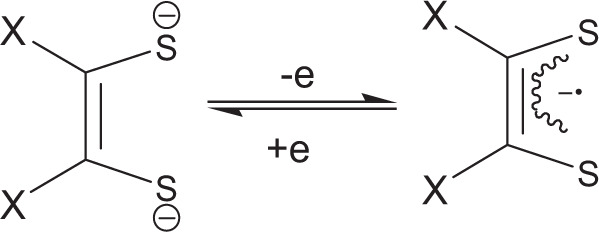
The redox reaction of nickel dithiolene. Reduction of nickel dithiolene via electron transfer reaction that converts it into the mono or dianionic form.

Here, we use nickel dithiolene as the active NIR material in the fusing agent because of its unique properties, and its ability to react with polar solvents containing a tert-amine (DMF, and N-methyl-2-pyrrolidone) which reduces it via electron transfer reaction, and converts it into the mono or dianionic form^18–23^ (M(RCSCSR)2-z, *z* = 1 or 2) as seen in Fig. 3.

The solubility and position of the absorption maximum of nickel bis(dithiolene) complexes is dependent on the solvent polarity and the nature of functional groups attached to the ligand. The reduction is accompanied by a shift in the absorption further into the near-IR region, with up to a 94 nm shift when the solvent is changed from toluene to DMF. A color change of the metal complex from green to red is observed upon reduction^12^. The color of the metal complex solution indicates the oxidation state of the bis(dithiolene) complex.

**Fig. 4:**
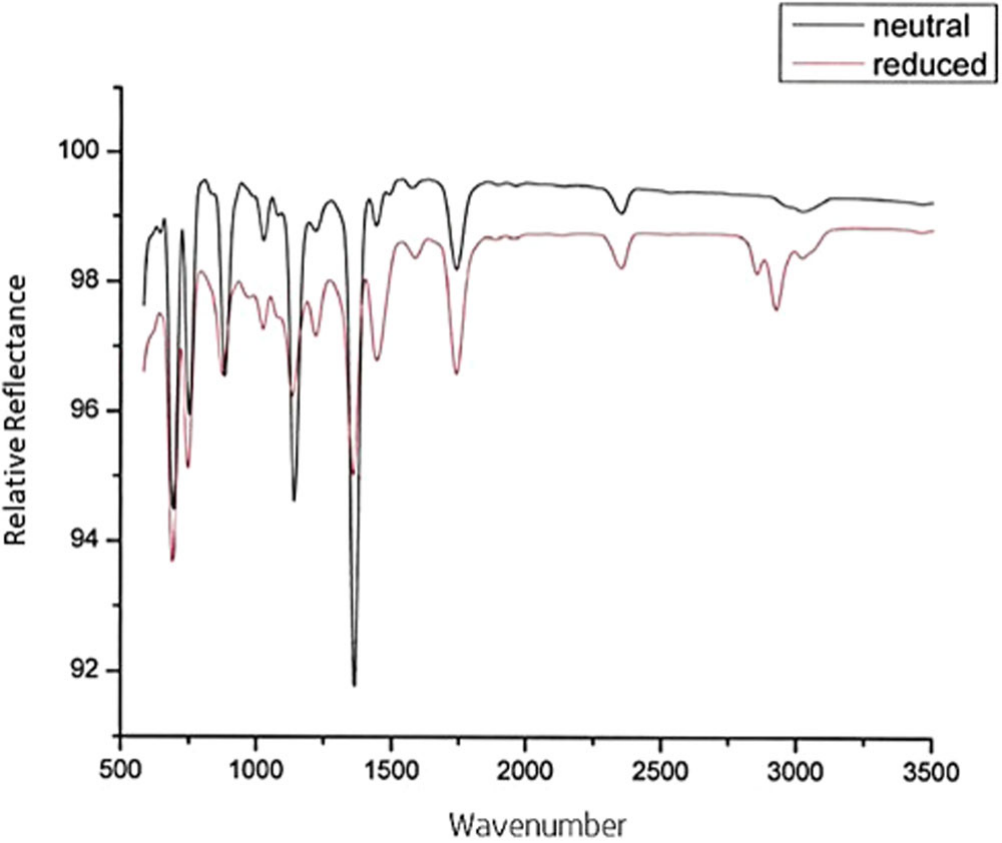
Overlaid FTIR of unreacted neutral vs. reduced and stabilized dithiolene. FTIR of the neat green and red isolated powder. The reduction reaction itself does not change the chemical structure other than adjusting the relative strength of bonds. However, the reduced form will react with tetraethyl ammonium bromide, precipitating it.

The red reduced form was isolated by a procedure published by Maki et al.^24^. A solution of 2.2 g. of tetraethylammonium bromide in 300 ml. of isopropyl alcohol–water (40% v./v.) was added to a solution of the red state (see ink formulation for formulation procedure). The red-brown crystals produced were collected, washed with water, and recrystallized from a mixture of acetone (10 ml) and isobutyl alcohol (25 ml.) by addition of 200 ml. of n-pentane; 3.9 g. (62%) of red-brown platelets separated by filtration and were washed with n-pentane and air-dried. Figure 4 compares the FTIR of the neat green and red isolated powder. The reduction reaction itself does not change the chemical structure other than adjusting the relative strength of bonds. However, the reduced form will react with tetraethyl ammonium bromide, precipitating it. This creates a new series of peaks around 3000 cm^−1^ associated with the aliphatic C–H from the attached ethyl group from the tetraethyl ammonium bromide.

### Ink formulation

The agent formulation contained 1 wt % green nickel dithiolene powder, which was reduced to the red state by heating at 100 °C for 10 min in a reducing solution, either a thiol dissolved in 2-pyrrolidone or a hindered secondary amine dissolved in 2-pyrrolidone.

**Fig. 5:**
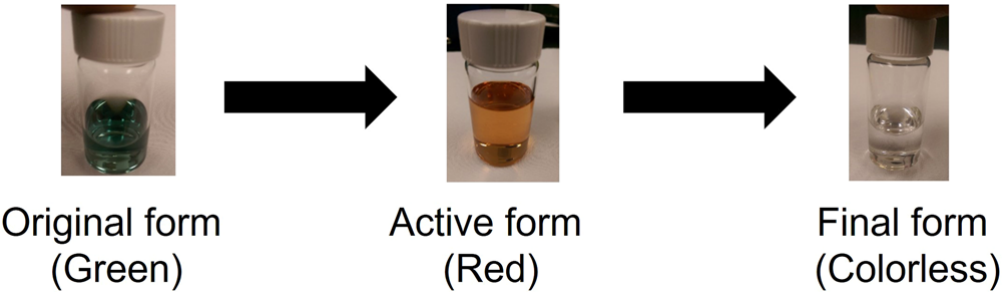
Reduction of nickel dithiolene from original green to red to colorless state. FTIR of the neat green and red isolated powder. The reduction reaction itself does not change the chemical structure other than adjusting the relative strength of bonds. However, the reduced form will react with tetraethyl ammonium bromide, precipitating it.

**Fig. 6:**
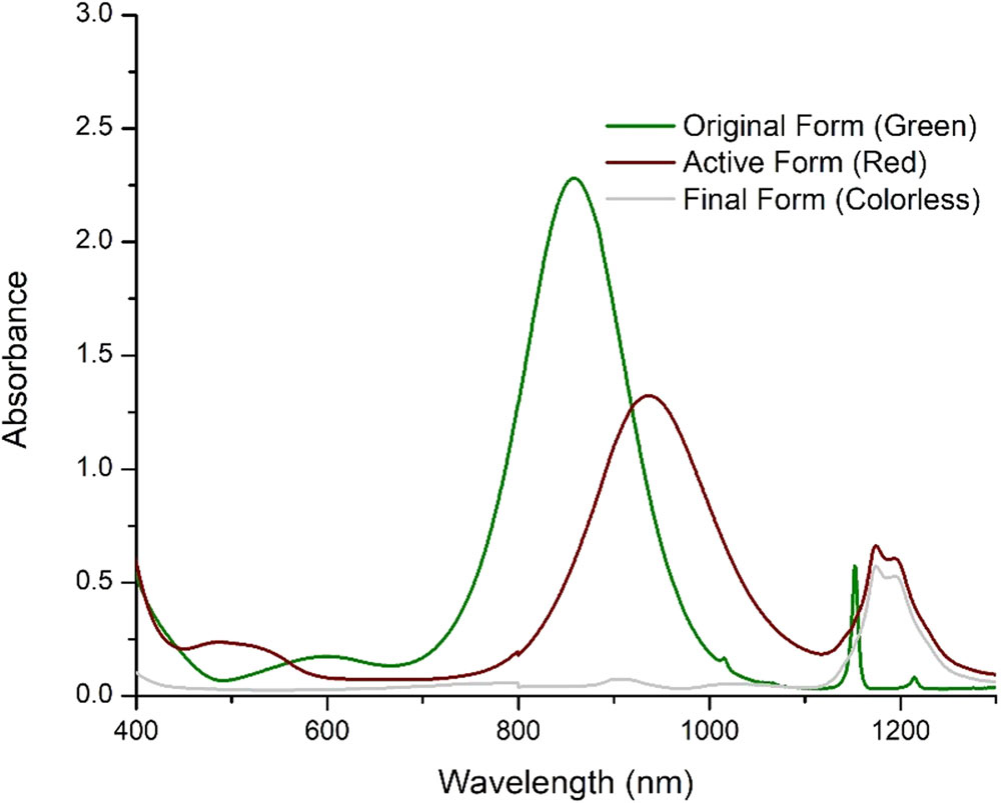
The UV–Vis-NIR absorption spectra of the nickel dithiolene. Green state, red state, and colorless state. A shift in the near-IR peak from 855 nm in the green state to 905 nm in the red state, followed by a complete disappearance of the IR peak when the red form is completely reduced to the colorless form.

After all the green powder was completely reduced to the red state, the stock solution was combined with HP’s proprietary ink vehicle to generate a thermal ink jet compatible formulation. The red state can further be reduced to colorless as shown in Fig. 5 by increasing the concentration of the reducing solution and/or by further heating, depending on the concentration of reduced nickel dithiolene in the solution. A shift in the near-IR peak from 855 nm in the green state to 905 nm in the red state was observed, followed by a complete disappearance of the IR peak when the red form is completely reduced to the colorless form see Fig. 6.

The red state can undergo a reversible oxidation to the green state when an oxidizing solvent like water or acetone is added. The colorless state is irreversible. The ink formulation is kept in the red state without oxidizing back to the green state or further reducing to the colorless state by carefully balancing HP’s proprietary ink vehicle to keep the amount of water in the formulation at 25%. This concentration was found to be ideal for maintaining the red state without the reaction shifting back to the green oxidized form or progressing to the colorless, fully reduced state. This amount was derived experimentally and remains stable for 6–8 months depending on storage conditions. The proprietary ink vehicle concentrate used is a preparation of various surfactants and solvents/humectants dissolved in water which are designed to deliver the correct physical properties for ink jet, e.g., surface tension, viscosity, and wettability.

**Fig. 7:**
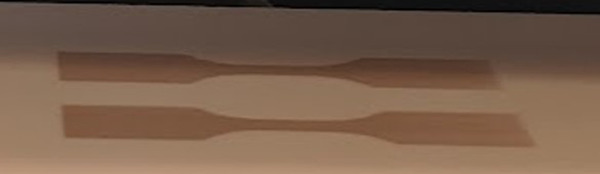
Red color of activating fusing agent visible on powder bed before fusing. X1 and X2 dog bones printed show the color of the AFA immediately after being deposited on the powder bed, before fusing and reduction turning colorless.

This balance is disrupted in our MJF printing process where the activated fusing agent in the red state is printed, harvests energy to fuse the build polymer powder into a layer of the 3D part, and then reduced to the colorless form after being exposed to 150 °C for 20 min. During the printing process, the loss of water from evaporation and the exposure to the polymer melt drives the reduction of the dithiolene to the final, colorless form. For the final formulation of the activated fusing agent, a nickel dithiolene concentration of 1% w/w was chosen. This concentration provides enough absorptivity to the layer for good fusion, while not loading so much dithiolene that it is unable to completely reduce by the end of the print, leaving a red tint to the parts. Figure 7 shows the color of the AFA immediately after being deposited on the powder bed, before fusing and reduction.

### 3D printing

**Fig. 8:**
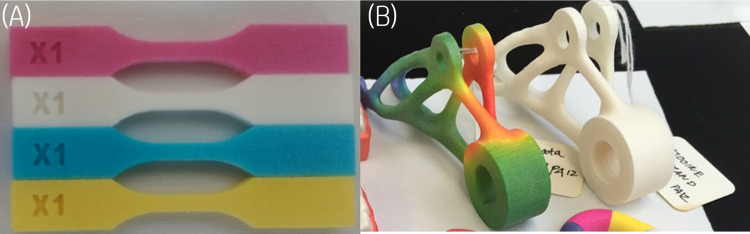
Colored and White tensile bars and stress analysis bracket printed with AFA. **A** Color ASTM type V dogbones. **B** 3D stress analysis bracket printed with AFA agent and PA-12 build material with only sandblast post processing. Colored parts printed with AFA when used with other colored inks.

The inkjet ink containing 1% w/w of the dye was printed to form parts with 0.1% w/w of the dye level ranging from 2 to 6 picolitre/600 dpi to the desired area that defines the layer of the corresponding 3D object. An IR halogen lamp with power between 500 and 750 W and color temperature between 2700 and 3400 K was then used as the light energy source to completely fuse the imaged areas with a 20–30 ips scanning speed during the fusing pass, after fusing, the residual heat of the build bed allowed the polyamide-12 to further reduce the red form of the nickel dithiolene in the ink to the colorless form, producing a white part having the native color of the powder. Other amide containing polymers were shown to perform similarly. When AFA is used with other color inks, the fused area will show the assigned colors as shown in Fig. 8.

**Fig. 9:**
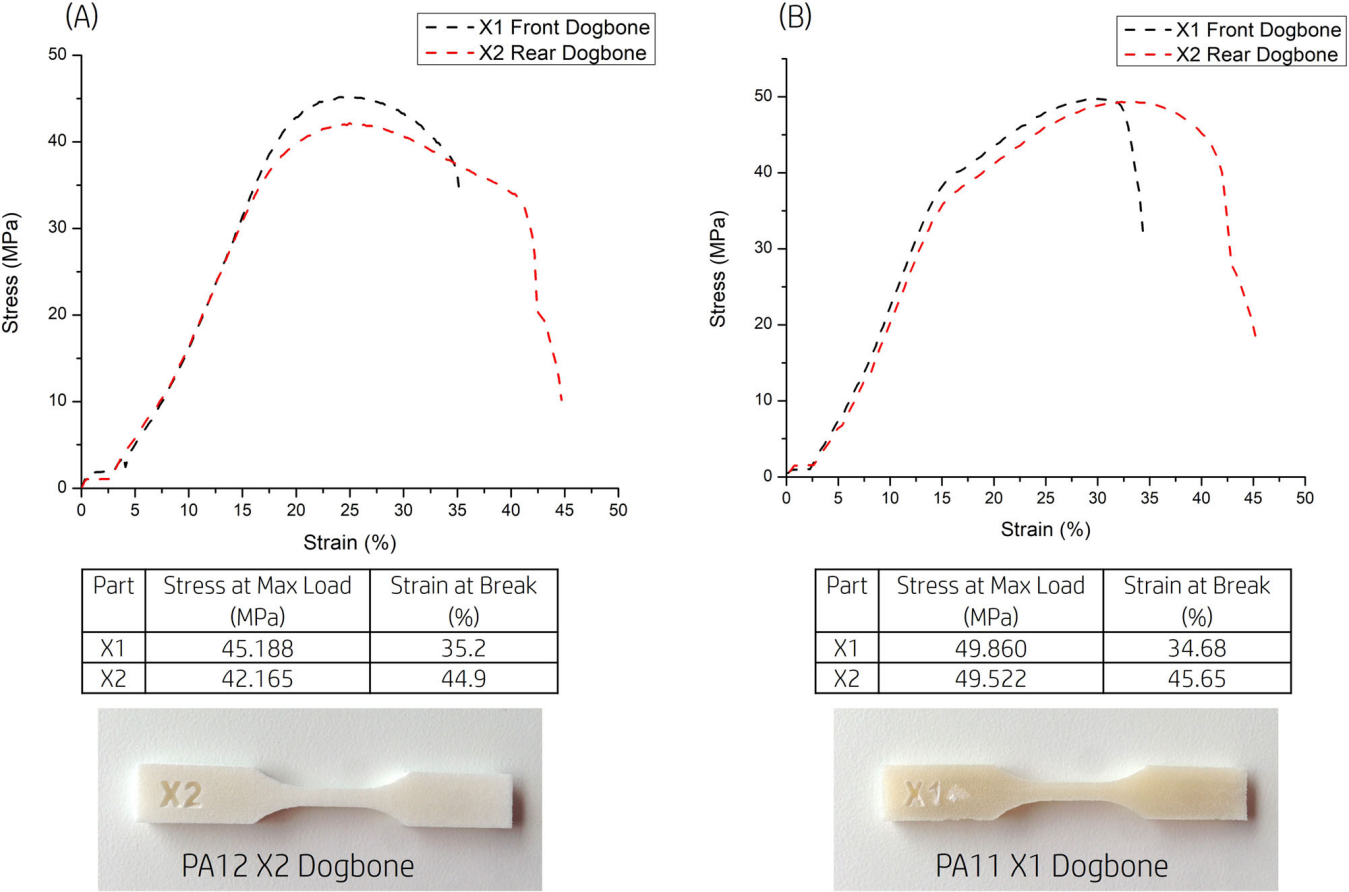
Mechanical properties of PA12 dog bone printed with AFA. **A** Stress–strain curve of PA12 *XY*-orientated ASTM D368 dog bone and corresponding X2 dog bone. **B** Stress–strain curve of PA11 XY orientated ASTM D368 dog bone and corresponding X1 dog bone. Stress–strain curves of PA12 and PA11 printed with AFA.

The stress–strain curve of *XY-*orientated ASTM D368 samples (dog bone) printed with AFA are shown in Fig. 9. Mechanical property comparisons for other printing technologies are shown in Table 1 including Stratasys J55 3D printer with photocuring Vero color material^25^ and 3D Systems ProJet CPJ color 3D binder jetting printer with VisiJet PXL material^26^. The thermal properties of the white and colored parts are similar to those of standard MJF parts published in the MJF white paper^27^.

**Table 1:** Mechanical properties of ASTM D368 samples printed with ProJet CPJ and J55 printers

Technology	UTS (MPa)	Elongation (%)	Density (g/cc)
ProJet CPJ	14.2	0.23	N/A
J55	40–55	5-20	1.17–1.18

## Discussion

Nickel Dithiolene based near IR materials can be reduced in the presence of common reducing agents like tert-amine (DMF, and N-methyl-2-pyrrolidone) via electron transfer reaction into the mono or dianionic form. The reduction of the dye allows formulation into a thermal ink jettable ink. The ink was utilized as a photothermal radiation absorber fusing agent in powder-based 3D printing. After absorbing energy turning it into heat, the red color bleaches and turns colorless to generate white and colored parts when used in combination with other colored agents.

## Methods

### Solvent and reagents

All chemicals were purchased from Sigma-Aldrich and were used as received unless otherwise stated. Nickel Dithiolene was purchased form Luminochem Budapest Hungary with particle size distribution *d*_90_ ≤ 50 μm, absorptivity in chloroform 30 L g^−1^ cm^−1^, absorption region λ = 820–950 nm see Fig. 6.

### Thermal inkjet testing

Inkjet-ability was tested using HP internal testing systems for microscopic drop imaging. The results showed no deflection of drops or build-up on the nozzle plate during firing. Drop weight and drop ejection velocity ranges between 2 and 50 nanograms and 5 and 15 m/s respectively for thermal inkjet print heads. These parameters are within the desired range for commercially viable formulations and indicate good performance and nozzle health.

### Polymer material

All the studies in this paper were conducted using HP 3D high reusability PA12 from HP Inc, Palo Alto, CA, USA.

### 3D printing

All printing in this work was performed using an internal advanced MJF print testbed, with the additional capability of printing more than two agents^28^. The production print process includes warming layers, 3D part printing layers, post printing layers, and safety cooling. By the time the build unit is ready to disconnect, the AFA has been at 150 °C for the required time to bleach the color from high irradiation absorbing red to colorless. The extracted parts are then sandblasted in a Powershot C (DyeMansion North America Inc., Austin TX, USA) for 15–20 min to remove most of the unmolten surface powder.

### Mechanical testing

Type V dogbone samples were printed for testing according to the ASTM D638 standard. Tensile tests were performed on these samples using a Instron 5969 dual column testing system (825 University Avenue Norwood MA USA 02062) with manual grips and 2kN load cell. A grip separation of 30 mm and strain rate of 3 mm/min were used. An Instron automatic video extensometer – model 2663 with 10 mm gauge length was used for measuring strain.

### Density measurements

The densities of Type V dogbone samples were measured using a Mettler Toledo XS205 Dual Range Balance using the Archimedes principle. Room temperature water was used as the auxiliary liquid for buoyancy measurements.

### UV–Vis measurements

UV–Vis spectra were recorded using a Varian Cary 6000i UV–Vis-NIR spectrophotometer with short-wave infrared (SWIR) 800–1800 nm range as well as the UV-visible 175–800 nm. Using narrow-band InGaAs detection and a 600 lines-per-mm diffraction grating for improved SWIR sensitivity. The samples were dissolved in chloroform or 2-pyrrolidone at 0.0001% w/w.

### FTIR measurement

Infrared measurements were performed on a Thermo Scientific NicoLET iS50 FTIR spectrometer (Waltham, MA, USA) from 3500 to 1000 cm^−1^ using an ATR diamond crystal.

## Data Availability

All relevant data has been supplied within the body of this paper.
